# The proportion of CD16^+^CD14^dim^ monocytes increases with tumor cell load in bone marrow of patients with multiple myeloma

**DOI:** 10.1002/iid3.53

**Published:** 2015-03-02

**Authors:** Anne M Sponaas, Siv H Moen, Nina B Liabakk, Emadoldin Feyzi, Toril Holien, Solveig Kvam, Lill Anny G Grøseth, Berit Størdal, Glenn Buene, Terje Espevik, Anders Waage, Therese Standal, Anders Sundan

**Affiliations:** 1 K.G. Jebsen Center for Myeloma ResearchTrondheim, Norway; 2 Department for Cancer Research and Molecular Medicine, Center for Molecular Inflammation ResearchNTNU, Trondheim, Norway; 3 Section of Hematology, St. Olav's University HospitalTrondheim, Norway

**Keywords:** Apoptosis, bone marrow, multiple myeloma, monocytes

## Abstract

Multiple myeloma is an incurable cancer with expansion of malignant plasma cells in the bone marrow. Previous studies have shown that monocytes and macrophages in the bone marrow milieu are important for tumor growth and may play a role in the drug response. We therefore characterized monocytes in bone marrow aspirates by flow cytometry. We found that there was significant correlation between the proportion of CX_3_CR_1_^+^, CD16^+^CD14^dim^ non classical monocytes, and percent plasma cells (PC) in the bone marrow of myeloma patients. The bone marrow monocytes could be stimulated by TLR ligands to produce cytokines which promote myeloma cell growth. The proportion of the non-classical monocytes increased with the tumor load, particularly in patients with tumor loads in the range of 10–30% bone marrow PC.

## Introduction

Multiple myeloma is a malignancy of plasma cells (PC), usually located to the bone marrow. Survival and proliferation of myeloma cells is critically dependent on the bone marrow microenvironment 1. Stromal cells including monocytes and macrophages can produce inflammatory cytokines such as IL6, which promote growth and increase survival of myeloma cells 2,3. In addition, macrophages and monocytes may produce anti-inflammatory cytokines that can promote tumor growth indirectly 3,4. In vitro, it has been shown that macrophages could support growth of myeloma cells and rescue them from chemotherapeutic drugs 5.

The bone marrow microenvironment is modified by the presence of malignant PCs. Thus, increased differentiation of monocytes into bone resorbing osteoclasts characterizes multiple myeloma 6. Furthermore, malignant PCs may modulate the numbers and composition of immune cells in the bone marrow. Monocytes and macrophages are central in inflammatory responses, and more macrophage/monocytes have been found in the bone marrow of myeloma patients compared with normal controls 7,8. It is however, not known which sub-types of monocytes are associated with myeloma disease.

Human blood monocytes can be classified into three distinct populations, classical CD16^−^CD14^+^ monocytes, intermediate CD16^+^ CD14^+^ and non-classical CD16^+^CD14^dim^ monocytes. The classical and intermediate monocytes are similar to the CCR2^+^ inflammatory mouse monocytes 9,10. In contrast, the CD16^+^CD14^dim^ cells are similar to CX_3_CR_1_^+^ mouse monocytes, which patrol blood vessels and respond to viral RNA and double-stranded DNA by producing granulocyte attracting mediators 11. Such cells have been associated with inflammatory disease such as rheumatoid arthritis and systemic lupus erythematosus (SLE) 11,12.

Here, we set out to characterize the monocyte sub-types in the bone marrow of a cohort of Norwegian myeloma patients.

## Results

### CD16^+^CD14^dim^ monocytes increase in the bone marrow of myeloma patients

**Figure 1:**
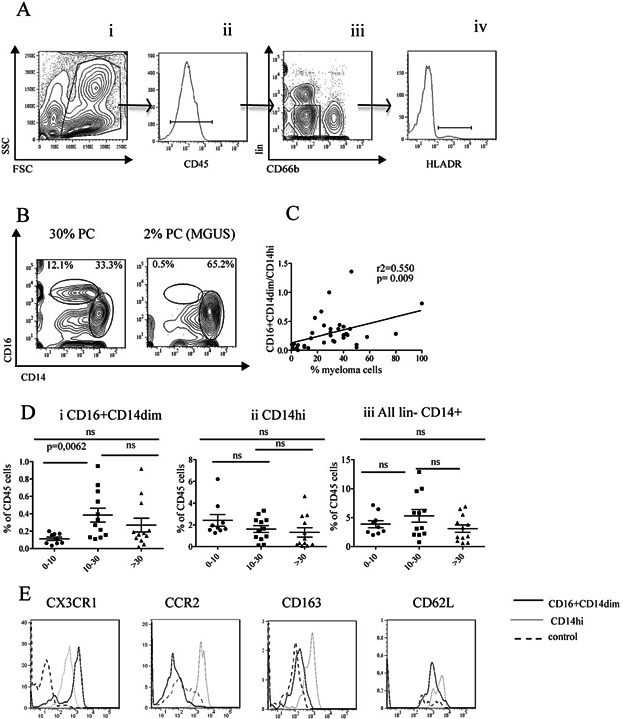
CD16^+^CD14^dim^ monocytes increase in the bone marrow of myeloma patients. (A) Gating strategy: Cells were stained with a cocktail of antibodies against CD66b, lineage (CD3, CD19, CD56, CD138, CD15, CD34, and CD235a), CD45, HLADR, CD16, and CD14 and analyzed on an LSR II Flow cytometer. Gates were set on FSC and SSC (i), doublets were excluded, and gates were set on the CD45^+^cells (ii). Lineage and CD66b^+^ were further excluded (iii). HLA DR^+^ cells are shown in (iv), and CD14 and CD16 profiles as shown in (B) were analyzed on these cells. (B) Representative CD14 and CD16 profiles from bone marrow aspirate from two patients with 30 and 2% PC, respectively. The gates indicated show the populations of % CD16^+^CD14^dim^ and CD14^high^ cells, respectively. (C) Correlation between the ratio of CD16^+^CD14^dim^ cells/CD14^high^ cells and percent PC in the bone marrow of patients (*n* = 33). Each dot represents a value from a patient as estimated by the gating indicated in Figure 1B. The *P*-value was calculated from Spearman's test. (D) CD16^+^CD14^dim^ monocytes increase in the bone marrow of patients with intermediate levels of 10–30% plasma cells. Bone marrow monocytes were stained and analyzed as described above and in Methods. Figures show monocyte populations as percent of CD45^+^ cells. Patients were grouped as low percent PC (0–10%) (*n* = 9), intermediate (10–30%) (*n* = 12), and high (>30%) (*n* = 12). Each dot represents a patient. Monocyte populations: (i) CD16^+^CD14^dim^, (ii) CD14^high^, and (iii) all monocyte populations (lineage^−^granulocyte^−^ CD14^+^). Statistical analysis was performed with Mann–Whitney Test. (E) CD16^+^CD14^dim^ cells express markers defining the CX_3_CR_1_ subpopulation of monocytes. Representative histograms of CX_3_CR_1_, CCR2, CD163, and CD62L expression on gated CD16^+^CD14^dim^ and CD14^high^ monocytes. Gates were set as described in (A). FMO (fluorescence minus one) is used as negative control.

In order to determine the sub type of monocytes present in myeloma patients, bone marrow cells from patients suffering from multiple myeloma were stained with a panel of antibodies against different monocyte subpopulations and analyzed by flow cytometry. The gating strategy is shown in Figure 1A. Gates were set on live cells with forward and side scatter (i), and on cells also expressing CD45 (ii). Lineage^+^ (CD3, CD19, CD138, CD56, CD15, CD34, and CD235a) and CD66b^+^ granulocytes were then gated out from the CD45^+^ cells (iii). The HLA DR profile within this gate is shown (iv). Plots of CD14 and CD16 expressing populations of the gated HLADR^+^ cells is shown in Figure 1B on cells from representative patients. The quantity of monocyte types was determined as a ratio of CD16^+^CD14^dim^/CD14^high^ cells (Fig. 1C) and as percentage CD16^+^CD14^dim^ cells of total CD45^+^ cells (Fig. 1D), respectively. The ratios of CD16^+^CD14^dim^/CD14^high^ cells increased with percent bone marrow PC, suggesting that more non-classical monocytes were present in bone marrow as the tumor mass increased (Fig. 1C). Similarly, the fraction of CD45^+^ cells that were CD16^+^CD14^dim^ was significantly higher in bone marrow from patients with 10–30% bone marrow plasma cells compared to patients with lower numbers of bone marrow plasma cells [Fig. 1D(i)]. Interestingly, patients with more than 30% plasma cells had variable amounts of CD16^+^CD14^dim^ cells, ranging from very low to high (Fig. 1D). No significant changes in the corresponding CD14^high^ population was observed [Fig. 1D(ii)].The proportion of non classical/classical bone marrow monocytes found in patients with low percent PC and their markers were similar to what was found in healthy controls (mean proportion: low percent PC: 0.0562+/−0.0011 and healthy controls: 0.08) 13. The bone marrow CD16^+^CD14^dim^ cells were similar to mouse patrolling monocytes as they expressed high levels of CX_3_CR_1_ and lower levels of CD163, CCR2, and CD62L than their CD14^high^ counterpart (Fig. 1E). We also found that high proportions of CD16^+^CD14^dim^ monocytes were present in the bone marrow and blood of the patients (Fig. S4), indicating that these cells circulate.

### The frequency of CD16^+^CD14^dim^ monocytes increases in patients with high amounts of apoptotic myeloma cells

**Figure 2:**
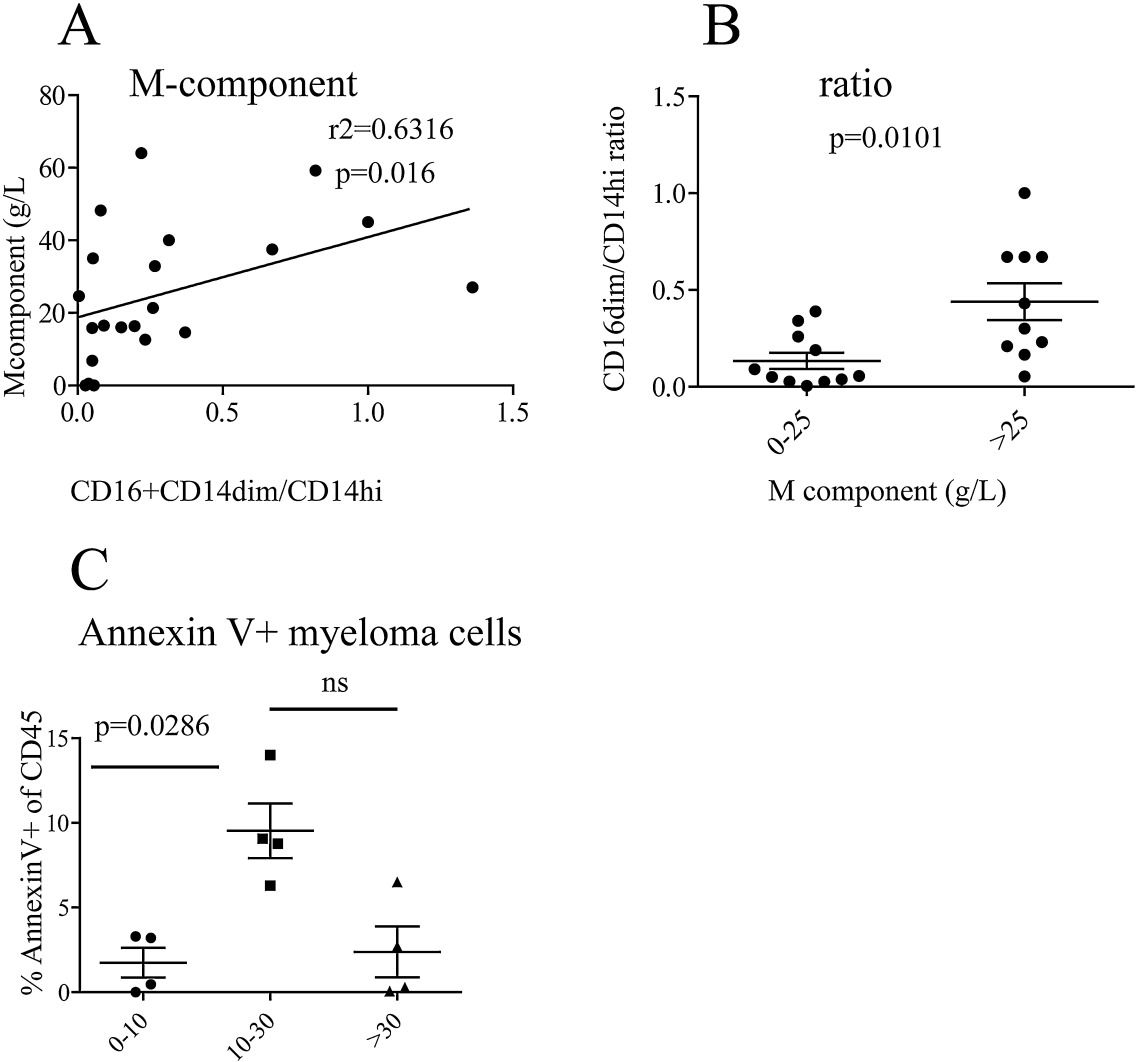
The frequency of CD16^+^CD14^dim^ monocytes increases with M-component and at high frequencies of apoptotic myeloma cells**.** (A) Increase in CD16^+^CD14^dim^ monocytes correlates with concentration of M component in serum. This figure shows the concentration of M-component in the patients’ serum versus the ratio of CD16^+^CD14^dim^/CD14^high^ cells. Each dot represents a patient (*n* = 22). *P*-value was calculated from a Spearman's test. (B) The proportions of CD16^+^CD14^dim^ monocytes were significantly higher in patients with high levels of M-component. Patients were grouped into two groups, below median value of 25 g/L (0–25 g/L, *n* = 12) and above (>25 g/L, *n* = 10), serum M-component. Each dot represents one patient and *P*-value was calculated from Mann–Whitney Test. (C) Patients with intermediate levels of bone marrow PCs had more apoptotic PCs. Bone marrow aspirate cells were stained with a cocktail of anti CD138, CD38, CD19, CD3, CD45, Annexin V, and 7AAD. Gates were set on live cells; duplet and lineage (CD3, CD19) were excluded. Gates were then set on the CD138^+^ CD38^+^ myeloma cells and the frequency of Annexin V^+^ 7AAD^+^ double positive cells of the CD45^+^ (high and low) population is shown. Patients were grouped as low percentage of bone marrow PC (0–10%) (*n* = 4), intermediate (10–30%) (*n* = 4), and high (>30%) (*n* = 3). Each symbol represents one patient and *P*-value was calculated using Mann–Whitney Test.

The expansion of the non-classical monocytes could be generated by several mechanisms. One explanation could be that the non-classical monocytes were expanded after stimulation with M-component produced by the myeloma cells. Immunoglobulins in the form of immune complexes have been associated with inflammatory diseases. In SLE for example, patients have high numbers of non-classical monocytes concomitant with elevated levels autoantibodies and immune complexes 11. As myeloma cells produce high quantities of immunoglobulins 10, we asked whether there was a correlation between the concentration of M-component in serum and proportion of CD16^+^CD14^dim^ cells in the bone marrow. A weak correlation was found (Fig. 2A, B). Furthermore, when the patients were divided into two groups, one group with M-component above the median level of all patients (i.e., 25 g/L), and one group below we also observed that patients with M-component above 25 g/L had significantly more CD16^+^CD14^dim^ monocytes (Fig. 2A, B) than the patients with low M-component. However, the M component levels and may only reflect tumor loads in the patients.

There could also be other mechanisms behind the increase of the non-classical monocytes in the myeloma bone marrow. It is proposed that sterile inflammation is one of the mechanisms behind symptoms of multiple myeloma 2, but the driving forces behind the inflammation are not known. However, it has been proposed that dying cancer cells could cause inflammation 2. Indeed, macrophage activation and sterile inflammation was seen in mice where DNA from apoptotic cells accumulated in macrophages 14. We therefore set out to investigate whether there was increased apoptosis of myeloma cells in the patients with elevated levels CD16^+^CD14^dim^ cells. In some patients, sufficient cells were available for staining with markers for cell death, and we detected significantly more apoptotic PC in patients with intermediate tumor load than in patients with less than 10 or more than 30% myeloma cells (Fig. 2C). We also found that the proportion of non classical monocytes increased with the proportion of apoptotic myeloma cells (Fig. S1).

### Bone marrow monocytes stimulated with TLR ligands produce myeloma growth factors

**Figure 3:**
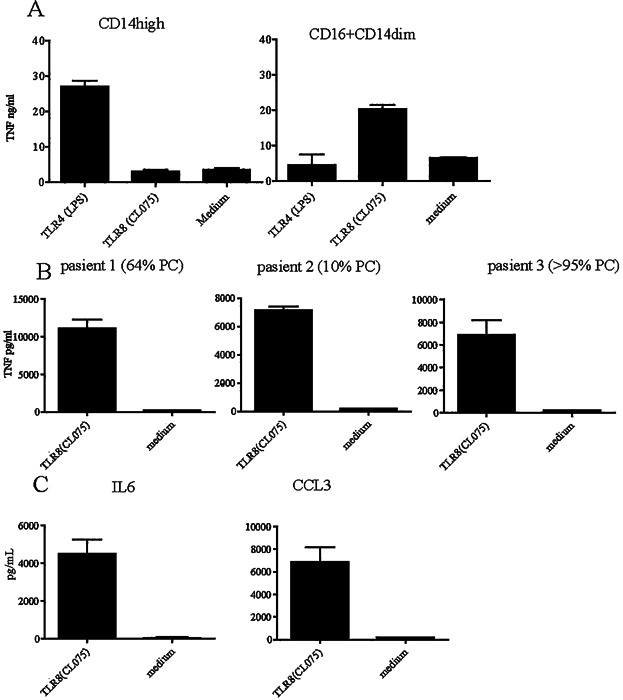
Bone marrow monocytes stimulated with TLR8 ligands produce myeloma growth factors. (A) 100 000 sorted CD14^high^ and CD16^+^CD14^dim^ blood monocytes were cultured with 100 ng/mL LPS, 1 μg/mL CL075 or medium alone for 12 h in 100 μL medium in a 96 well plate. TNFα was measured in the supernatant by ELISA. (B,C) CD16^+^CD14^dim^ bone marrow monocytes from myeloma patients produce cytokines after stimulation with TLR8 agonist. Monocytes were immunomagnetically purified from bone marrow of myeloma patients as described. A total of 200,000 cells were stimulated with 1 μg/mL TLR8 agonist (CL075) or medium for 24 h before harvesting the supernatant, (B) TNFα secretion in the supernatant of monocytes from three representative patients out of eight analyzed. The monocytes were enriched from bone marrow as described in the Materials and Methods and stained with anti CD16 and anti CD14 after enrichment (patient 1: 9.3% CD16^+^CD14^dim^, patient 2: 10.3% CD16^+^CD14^dim^, patient 3: 13.8% CD16^+^CD14^dim^). (C) CD16^+^CD14^dim^ monocytes produce myeloma growth factors after TLR8 stimulation. (D) IL6 and CCL3 in the supernatant from one representative patient out of eight analyzed. Cytokines were detected using Multiplex (27 plex BioRad). Figures shows mean and SEM of triplicate wells from each patient analyzed (D).

As we found that there were more apoptotic myeloma cells in the patients with high levels of non-classical monocytes, we hypothesized that this expansion could be a result of an inflammatory response generated by nucleic acids released from dying myeloma cells. The non-classical monocytes had the potential to respond to nucleic acids since we as well as others 15, detected toll-like receptor (TLR) 8 expression on these cells (data not shown). We also set out to test whether the non-classical monocytes could be stimulated to produce inflammatory cytokines by TLR8 agonists. As insufficient bone marrow aspirate was available to sort and stimulate the two different monocyte populations separately, we relied on the observations that the two populations respond to different TLR ligands 15. Indeed, as reported by Cros et al., we confirmed that only non-classical blood monocytes responded to the base analogue TLR8 ligand CL075 and not to LPS 11, whilst, the classical CD14^high^ blood monocytes only responded to the TLR4 ligand LPS and not the TLR8 ligand (Fig. 3A). Stimulation of the monocytes enriched from the bone marrow of myeloma patients with the TLR8 ligand CL075 induced TNF α production (Fig. 3B). Indeed, we found that non classical monocytes produced growth factors that could support myeloma cells (Fig. S3b).Taken together, the data shown in Figure 2A and B indicates that the non-classical monocytes from the patient's bone marrow have the potential to be stimulated by nucleic acids. Other inflammatory cytokines known to support myeloma growth, such IL6 and CCL3 (Fig. 2C) were also secreted into the supernatant after stimulation of the bone marrow monocytes with the TLR8 agonist. The classical monocytes could also produce inflammatory cytokines since LPS treatment of enriched bone marrow monocytes resulted in TNFα, IL6, and CCL3 production (Fig. S2). Thus, both classical and non-classical monocytes were present among the purified bone marrow monocytes. However, taken together, the results indicate that the CD16^+^CD14^dim^ monocytes may produce inflammatory cytokines that could support myeloma growth in response to TLR8 ligands**.** We failed to detect IL10 mRNA or mRNA coding for any other anti-inflammatory cytokines in monocytes enriched from the bone marrow and cultured with mCSF (Fig. S5) or from monocytes sorted directly from the bone marrow (data not shown).

## Discussion

We found in this study that the proportion of non-classical, CD16^+^CD14^dim^ monocytes is small in patients with normal to low bone marrow levels of PC (i.e., approx. 2% PC), and that the amount of these monocytes increases with tumor load in myeloma bone marrow (Fig. 1D). In contrast, we did not observe any significant change in the CD14^high^ monocyte population (Fig. 1D). The variation in monocyte levels seen in patients with high tumor load could be because the proportion of monocytes of the CD45^+^ population will drop as the number of CD45^+^ PC increase. Alternatively, there could be a disruption in hematopoiesis in patients with high tumor loads 16,17. Patients with advanced disease and high tumor load often present with anemia, leukopenia, and thrombocytopenia of indicative of defective hematopoiesis 18. At the late stage of the disease, the tumor is often not dependent on growth factors or the bone marrow milieu 19 and may therefore not require the presence of macrophages/monocytes in the bone marrow. There were more of the non-classical monocytes CD16^+^CD14^dim^ in patients with apoptotic myeloma cells. These could be stimulated with TLR8 ligands implying that they are functional monocytes and that they can produce cytokines in response to certain stimuli that may promote myeloma growth. Indeed, we found that supernatant from sorted non classical monocytes were able to support the growth of the myeloma cell line INA6 (Fig. S3b) suggesting that these cells could support the growth of myeloma cells in patents.

The expansion of the non-classical monocytes could be generated by several and non-mutually exclusive mechanisms. Even in cases where there are well-established correlations between non-classical monocytes and certain diseases, we do not know directly why that is the case, although it has been suggested that immune complexes or nucleic acids stimulate non-classical monocytes to expand and produce inflammatory cytokines and sterile inflammation.

It is proposed that sterile inflammation is one of the mechanisms behind symptoms of multiple myeloma 2 and this could lead to the expansion of the non-classical monocytes after stimulation with TLR ligands. Thus, it has been proposed that dying cancer cells could cause this inflammation 2. Indeed, is was shown in mouse models that macrophage activation and sterile inflammation was induced where DNA from apoptotic cells accumulated in macrophages 14. In line with this, we found an elevation of TRAIL and Lymphotoxin concentrations in the bone marrow of myeloma patients compared with controls in another cohort of patients (unpublished results) indicating that cell death and inflammation takes place. Thus, our data could suggest that components from apoptotic cells were responsible for stimulating the CD16^+^CD14^dim^ population in the myeloma patients. This is supported by the observation that increased IL1Rα and MIP1α, which has been associated with activation of CX_3_CR_1_ monocytes, were raised in the bone marrow of myeloma patients compared with healthy controls 20.

The relationship between the classical and non-classical monocyte populations is not fully known, although in mice, the CCR2^+^, inflammatory monocytes and the CX_3_CR_1_^+^ patrolling monocytes are proposed to develop from different precursors. It is, however, also possible that one population could develop into the other during inflammatory conditions 9. In humans it has been reported that CD16 is up-regulated in classical monocytes by TGFβ1 production after activation 21. Myeloma cell produce high levels of TGFβ1 (reviewed in 22, that potentially could up-regulate CD16 expression. Thus it is possible that CD14^high^ monocytes developed into the CD16^+^CD14 ^dim^ cells in the patients in the presence of increased levels of PC. Alternatively, CD16^+^CD14 ^dim^ cells could migrate to the bone marrow from the circulation to areas of cell death.

Increased apoptosis in the myeloma cells from the patients with high ratio of CD16^+^CD14^dim^ cells, suggests the presence of myeloma cell derived ligands such as cellular DNA and RNA. However, the presence of such ligands has to our knowledge not yet been identified in bone marrow aspirates of myeloma patients. It is however possible that apoptotic cells and DNA or RNA from apoptotic cells are rapidly removed by macrophages adjacent to the dying myeloma cell and will therefore not easily be detected.

In recent years, it has become evident that the micro environment is very important not only in the oncogenesis of myeloma, but also in the clinical presentation and response to treatment. Monocytes, macrophages, and DCs are very important components of the myeloma environment. Classical monocytes stimulated by myeloma derived products and plasmacytoid DC 23 stimulated with mitochondrial DNA released from dying myeloma cells for example could support myeloma growth. In addition, our observation that the non-classical monocytes are present at a higher proportion in patients with apoptotic myeloma cells, suggest that these cells could be one of the cells types contributing to the disease process. However, further experiments are required to determine the mechanism behind the increase of non-classical monocytes in multiple myeloma and how they contribute to the disease.

## Materials and Methods

### Patients

Bone marrow cells were collected in Sodium Heparin (Wockhardt, Wrexham, UK) from the pelvis of monoclonal gammopathy of unknown significance (MGUS), newly diagnosed and relapsing myeloma patients. The patients were enrolled after informed consent and the study was approved by the Regional Ethics Committee (REK 2011–2029). Percentage bone marrow PC and serum M-component were determined as part of standard diagnostic procedures. Two patients out of the 33 analyzed had moderately elevated CRP (>5 g/L), but without indication of infection. Patients were divided in three groups with similar age and sex distribution (Table1). The fraction of PC, median age and age range among the three groups were: 0–10% PC [62 years, range from 44 to 72 (5 male, 4 female)] 10–30% PC (64.5 years, range from 50 to 84 (6 male, 6 female)], >30% PC [72 years, range from 53 to 86 (6 male, 6 female)]. The analysis was done on freshly collected samples.

**Table:** Patients

Group (% plasma cells)	No (total = 33)	Median age** (range to-from)	sex
0–10	9	62 (44–72)	5m, 4f
10–30	12*	64,5 (50–84)	6m, 6f
>30	12*	72 (53–86)	6m, 6f

* One patient with moderately elevated CRP values of >5 g/L; but without indication of bacterial or viral infection.

** Age at sampling.

### Reagents and antibodies

Anti-human CD66bFITC, CD3, CD19, CD14, CD163,CD138, CD56, CD15 all PE conjugated, HLADRV450, CD45V500, CD16APC-H7, CD14PE-Cy7, CD38PE-Cy7, CD 206APC were obtained (BD Pharmingen, Stockholm, Sweden). CD34PE, CD235aPE, CD11bPerCp-eFlour710 (eBioscience, San Diego, CA, USA), CD192APC, and CX3CR1AlexaFlour647 were from BioLegend, San Diego, CA, USA. Human FcReceptor binding inhibitor, 7AAD, and Annexin V-Alexa647, were obtained from eBioscience, San Diego, CA, USA, Sigma–Aldrich, (St. Louis, MO, USA), and Molecular Probes, Eugene, OR, USA, respectively.

### Flow Cytometry

Bone marrow cells were analyzed after RBC lysis in hypotonic buffer (dH_2_O, NH_4_Cl, NaHCO_3_, EDTA). A total of 0.5 million cells were stained with a cocktail of antibodies against human CD66b, lineage (CD3, CD19, CD56, CD138, CD34, and CD235a), CD45, HLADR, CD16, and CD14 for 30 min on ice after 20 min incubation with human Fc Receptor binding inhibitor. Flow cytometry was performed using LSR II (Becton Dickinson, Franklin Lakes, NJ, USA) with FACS Diva software (Becton Dickinson). Samples were analyzed with FlowJo 7.6 (TreeStar, Ashland, OR, USA). For the monocyte population gates were set on live cells with forward and side scatter and duplets were gated out. Lineage and CD66b^+^ granulocytes were excluded and the CD14 and CD16 were determined on the CD45^+^, HLA-DR^+^ cells. Myeloma cells were stained in a separate panel with CD3, CD19, CD138, CD38, CD45, Annexin V, and 7AAD. Gates were set on FSC and SSC and CD45^+^ cells were included whilst lineage CD3^+^ and CD19^+^ cells were excluded. More than 95% of the plasma cells in the patients expressed CD45. % Annexin V and 7AAD double positive cells were determined on all CD38^+^ CD138^+^ cells.

### Cell sorting of monocytes from blood and bone marrow

RBC lysis of peripheral blood was performed using a hypotonic buffer before sorting of monocytes into CD16^+^CD14^dim^ and CD14^high^ populations on FACS Aria (Becton Dickinson) after staining with a cocktail of antibodies against human CD66b, lineage (CD3, CD19, CD56, CD138, CD34, and CD235a), CD45, HLADR, CD16, and CD14 for 30 min on ice. Before staining with antibodies the cells were incubated for 20 min with human FcReceptor binding inhibitor. 7AAD (Sigma–Aldridge, St. Luis, MO, USA) positive, dead cells and duplets were excluded. Sorting gates were set as described in Figure 1. More than 95% purity was achieved for both populations.

### Enrichment of bone marrow monocytes by immunomagnetic purification

Bone marrow monocytes were purified using a pan monocyte isolation kit (Miltenyi Biotech, Bergisch Gladbach, Germany) after density gradient centrifugation (Lymphoprep, Axis-Shield, Oslo, Norway). This kit removes CD3, CD19 positive cells. In addition we removed the CD66b and CD56 positive cells adding biotinylated antibody. Figure S1 shows CD14 expression on the purified cells from a typical experiment. 90% or more of the cells expressed CD14. Flow cytometric analysis showed that both CD16^−^CD14^high^ classical and CD16^+^CD14^dim^ non classical monocytes were after the purification (data not shown).

### Stimulation of monocytes

A total of 100,000 sorted blood monocytes or 200,000 immunomagnetically purified bone marrow monocytes were cultured in RPMI 1640 (Sigma–Aldrich, Schnelldorf, Germany) supplemented with L-glutamine (100 μg/mL), gentamycin (20 μg/mL) and 20% heat-inactivated fetal calf serum (FCS). The cells were stimulated with 100 ng/mL lipopolysaccharide (LPS) (InVivogen, San Diego, CA, USA) or 1 μg/mL CL075 (InVivogen) for 12 h in 100 μL in a 96 well flat bottomed tissue culture plate (Sigma-Aldridge, Costar). Cytokines were measured in the supernatant by Mulitplex or ELISA.

### Cytokine assays

Cell culture supernatants or bone marrow plasma (stored at −80°C) were analyzed for cytokines using Bioplex Precision Human Cytokine Assay (27plex and 23plex assay, BioRAD, Hercules, CA, USA) or ELISA (R&D Systems, Minneapolis, MN, USA) following the supplier's protocol.

### Statistical analysis

Tests were performed using Graph Pad Prism 5 software. Correlations were determined using Spearman's Test. Comparisons between groups were done with Mann–Whitney. Significance was determined as *P* < 0.05.

## Supporting Information

**Fig. S1**. Correlation between the ratios of CD16^+^CD14^dim^ cells/CD14^high^ cells and percent apoptotic plasma cells in the bone marrow of patients (*n* = 1). **Fig. S2**. Purified monocytes were stained with antibodies against CD16 and CD14 and analyzed on LSR II Flow cytometer (BD Biosiences). **Fig. S3a**. CD16-CD14^high^ bone marrow monocytes from myeloma patients produce cytokines after stimulation with TLR4 agonis. **Fig. S4**. Similar proportions of non classical monocytes in the blood and bone marrow. Blood and bone marrow from patients were stained with the same panel of antibodies as described in Figure 1. **Fig. S5.** Purified bone marrow cells were enriched as described in Materials and Methods.
