# Supplementary material for: The proportion of CD16+CD14dim monocytes increases with tumor cell load in bone marrow of patients with multiple myeloma
Source: Immun Inflamm Dis. 2015 Mar 2;3(2):94–102. doi: 10.1002/iid3.53 (PMC4444152; doi:10.1002/iid3.53)
Supplement: Supplementary file 1 [file iid30003-0094-sd1.docx]

Supplementary Methods

**Nanostring analysis**

**50 ug RNA was used for the analysis. Samples were analyzed for gene expression using the Nanostring nCounter® GX Human Immunology v2 Kit (cat.no GXA-­‐HIM2-­‐24, NanoString Technologies, Seattle, USA). The nCounter Analysis System; consisting of the nCounter Prepstation  and nCounter Digital Analyzer was used with the manufacturer’s protocol. The nSolver analysis software was used to analyze the acquired data. Data was normalized on negative controls and several housekeeping genes**.

Supplementary Legends

**Fig S1**

**Correlation between the ratios of CD16^+^CD14^dim^ cells/CD14^high^ cells and % apoptotic plasma cells in the bone marrow of patients (n=12). Each dot represents a value from a patient. The ratios were determined by the staining and gating shown and described in in Figures 1A, B and Materials and Methods and Figure 2B. The p-value was calculated from Spearman’s test**.

Fig S2

Purified monocytes were stained with antibodies against CD16 and CD14 and analyzed on LSR II Flow cytometer (BD Biosiences). Gates were set on FSC and SSC as well as doublets. Histogram shows CD14 profile of a representative patient sample of 6 analyzed. % depicts proportion of CD14 + cells. The patient samples had varying percentages of CD16^+^CD14^dim^ cells (data not shown).

Fig S3

a.CD16^-^CD14^high^ bone marrow monocytes from myeloma patients produce cytokines after stimulation with TLR4 agonist. Monocytes were immunomagnetically purified from bone marrow of myeloma patients as described. 200,000 cells were stimulated with 100ug/mL LPS or medium for 24 hrs before harvesting the supernatants. Cytokines were detected using Multiplex (27 plex BioRad). Figures shows mean and SEM of triplicate wells from each patient analyzed. Figure shows TNFα, IL6 and CCL3 in the supernatant of a representative patient out of 8 analyzed.

**b.CD16^+^CD14^dim^ monocytes produce factors that support the growth of INA6 myeloma cells. CD16^+^CD14^dim^ monocytes were sorted from the blood of a healthy donor to a purity of > 95%. 10 000 cells were cultured with or without 1 ug/mL CL075 and the supernatant harvested after 12hrs. 10 000 INA6 cells were cultured with 10% medium from the cultured monocytes or with or without 1ng/ml recombinant IL6 for 3days. The proliferation was determined with CellTiter-Glo kit (Promega, Madison, USA) using the protocol provided by the manufacturer.**

**Figure S4**

**Similar proportions of non classical monocytes in the blood and bone marrow. Blood and bone marrow from patients were stained with the same panel of antibodies as described in Figure 1. Figure shows plot of CD16 and CD14 expression on cells gated as described in Figure 1.Patient A: 30% PC, patient B.: 10%(B) The gates indicated show the populations of % CD16^+^CD14^dim^ and CD14^high^ cells, respectively**. **The table below shows % cells of CD45+ cells**

**Figure S5**

**Purified bone marrow cells were enriched as described in Materials and Methods. 100 000 enriched cells containing both monocyte populations were cultured with 100 ng/mL recombinant mCSF ( PeproTech, Rocky Hill, USA), for 7 days before harvesting RNA (Qiagen,Hilden, Germany) and performing Nanostring analysis.**

**
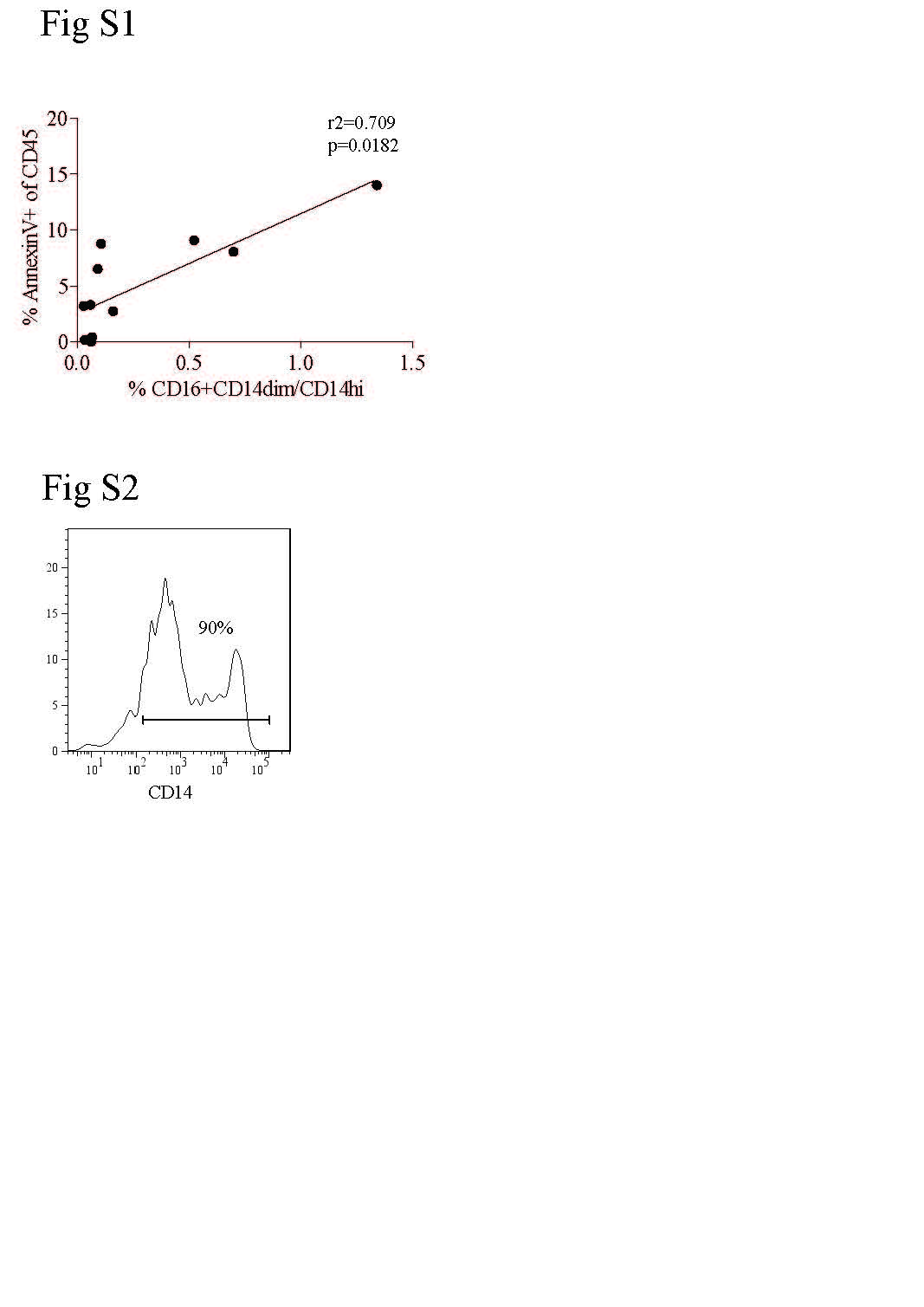
**

**
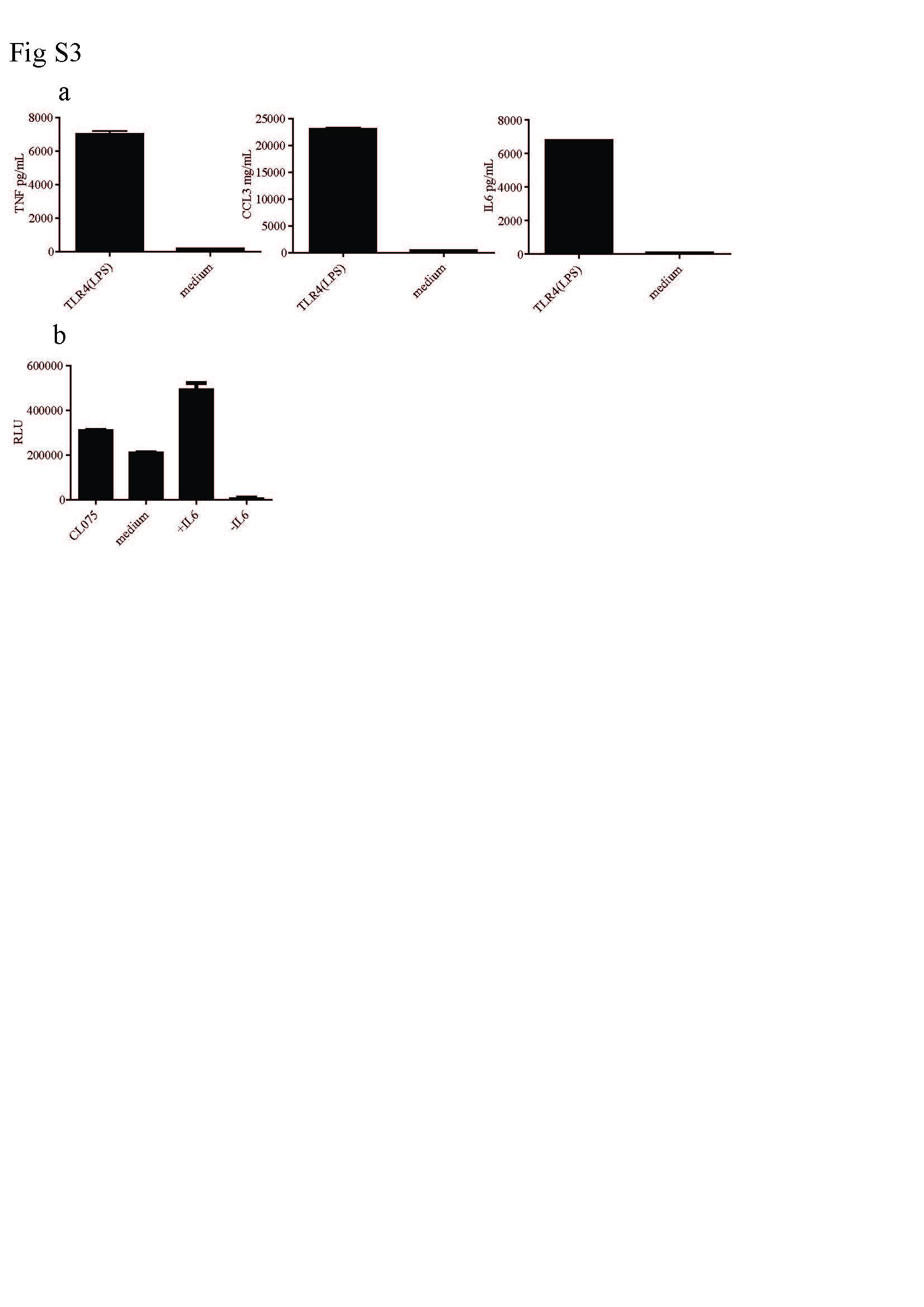
**

**
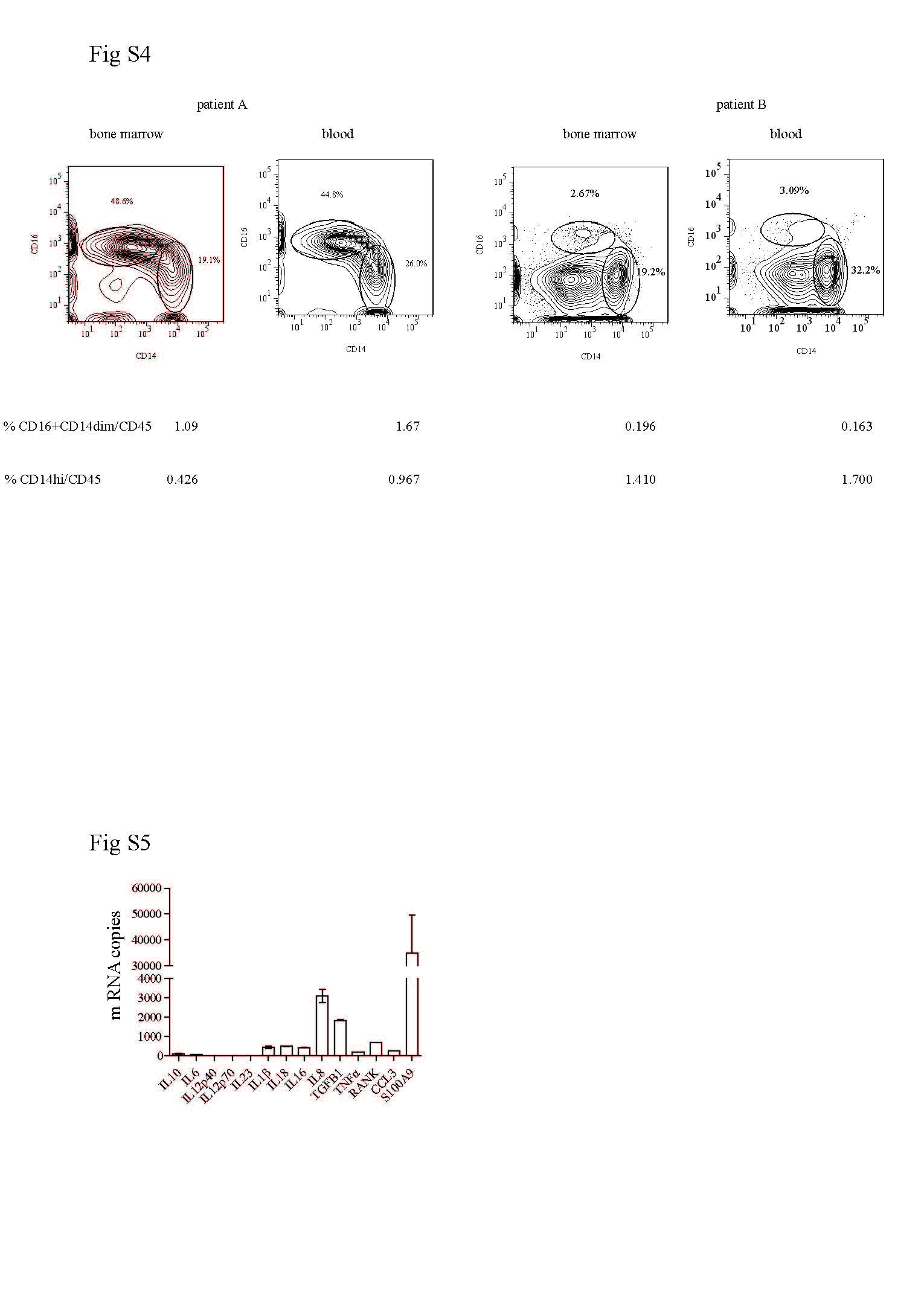
**
